# Combining Thermal Loading System with Acoustic Emission Technology to Acquire the Complete Stress-Deformation Response of Plain Concrete in Direct Tension

**DOI:** 10.3390/ma14030602

**Published:** 2021-01-28

**Authors:** Rui Zhang, Li Guo, Wanjin Li

**Affiliations:** Jiangsu Key Laboratory of Engineering Mechanics, Department of Engineering Mechanics, School of Civil Engineering, Southeast University, Nanjing 210096, China; ruizhang-nanking@seu.edu.cn (R.Z.); wjli2019@seu.edu.cn (W.L.)

**Keywords:** concrete, direct tension test, acoustic emission, thermal loading system, complete tensile stress-strain curve

## Abstract

The tensile properties of plain concrete are very important for the concrete structural design, and the complete tensile stress-strain curve is essential for creating accurate and reliable designs, especially when considering special load cases such as earthquakes and impacts. To study the complete tensile stress-deformation response of plain concrete, the direct tension tests were conducted on a novel thermal tensile testing machine (TTTM), which was reformed from a hydraulic universal testing machine (UTM). Acoustic emission (AE) technology was applied to monitor the damage process of plain concrete in tests. The TTTM was powered by the thermal expansion of loading columns, and had a stiffness similar to the specimen, thus eliminating the potential AE noises in the UTM, and simulating the rapid fracture process in real concrete structures. A static-dynamic acquisition system was established to obtain the complete tensile stress-strain curves, of which the data before and at the fracture moment were respectively acquired by the static acquisition system and the dynamic acquisition system. The AE technology is a useful approach to analyze the damage process of concrete, and makes it feasible to determine the damage state and the fracture location of the specimen in real time.

## 1. Introduction

The tensile properties of concrete materials are indispensable for the assessment and design of concrete structures. The complete tensile stress-strain curve of plain concrete, especially the softening response, is necessary when accessing the structural safety of concrete buildings subjected to various load cases. Meanwhile, the concrete materials bear the main load in the low reinforced or plain concrete structures, and the structural stiffness and stability may vary greatly once the concrete materials crack due to tension. Consequently, studying the complete tensile stress-deformation response of concrete is of significance in concrete structural engineering.

The tensile softening response of concrete is the most difficult part to acquire in the complete tensile stress-strain curve. The reason for this is that the concrete, as a quasi-brittle material, is easy to fall into an unstable cracking state when the energy released by concrete structures or test machines is greater than the energy material needed to crack stably. The extremely short time of the unstable cracking process make it difficult to record the relevant data. At present, the common test methods to study the tensile softening response of concrete can be mainly classified into three categories: direct tension tests (DTTs) [1,2,3,4,5]; three-point bending beam tests (TPBBTs) [2,6,7]; and splitting tensile tests (STTs) [8,9,10,11]. Compared with the TPBBT and the STT, the softening response of specimens in the DTT is more difficult to observe. The DTT, however, is a better approach to obtain the complete tensile stress-strain curve and study the damage evolution of concrete in tension. This is because both the TPBBT and the STT are indirect tensile tests, in which stress distribution is more complex and keeps changing with the increasing damage. Moreover, in the TPBBT and the STT, the complete tensile stress-strain curves are obtained through the inverse analysis method [2] and theoretical analysis model [10], respectively, which may cause the reliability of the test results to be affected by the relevant parameters.

Since Evans and Marathe [12] observed the softening response of plain concrete in a DTT and obtained the corresponding complete tensile stress-strain curve, there has been an outpouring of literature focused on the tensile properties of various concrete materials [2,13], the effects of different test methods [3,4,5,14,15,16] and the simulations of the fracture process [17,18,19]. Generally, the DTTs are conducted on a tensile test machine (TTM) with high stiffness [3,14], or a special electronic servo-controlled TTM with a fast response time [4,20,21]. For the former test machine, it is difficult to build the hydraulic control system with high precision and excellent dynamic performance [20,22], while for the latter, it is hard to increase stiffness of the load frames to an adequate degree. Additionally, in the DTTs conducted on the two above-mentioned test machines, the softening response data of concrete is obtained under slow loading conditions. This slow loading condition, however, may not be realistic in the fracture process of concrete structures. In reality, the release of the elastic energy stored in the uncracked part of concrete structures might cause a sudden change of load and deformation, and consequently trigger an unstable cracking process. Unfortunately, the unstable cracking process is common in the tests of low reinforced concrete members [23], fiber-reinforced polymer reinforced concrete members [24] and fiber-reinforced concrete with low fiber content [25,26]. Correspondingly, new further measure strategies are needed to study the rapid damage process in the post-peak stage of concrete materials.

**Figure 1:**
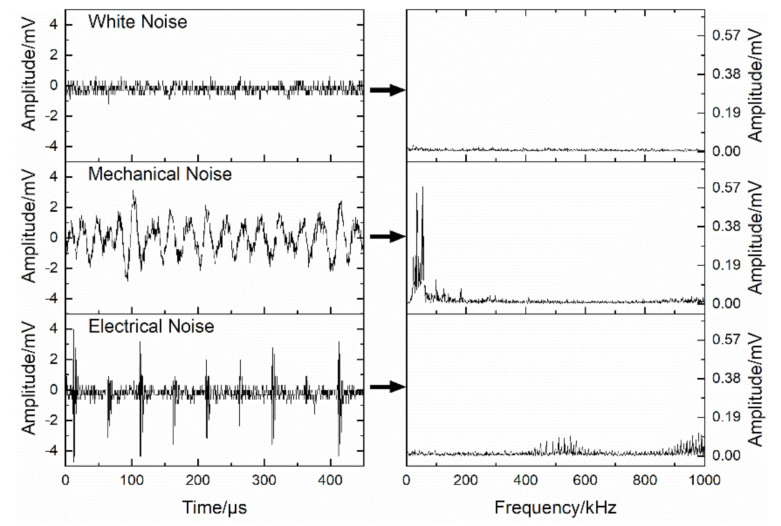
The waveforms and Fourier transform diagrams of acoustic emission (AE) noises observed in the pretests conducted by the authors on a hydraulic universal testing machine (UTM).

In addition, due to the inhomogeneity of concrete, the damage process of the concrete material in DTTs is complex and unpredictable. Consequently, the real-time damage monitoring can facilitate the analysis of the cracking process, and guide the adjustment of the instruments during the tests. Acoustic emission (AE) technology (PCI-2 Based System, Physical Acoustics Corporation, Princeton Junction, NJ, USA), as a useful method to analyze the damage evolution inside the concrete material in real time [27], was commonly applied in DTTs [4,5,28]. However, AE monitoring in the DDTs conducted on electro-hydraulic servo universal testing machines (UTMs) may be affected by the AE noises generated in the operation of UTMs. The hydraulic pump can be regarded as a vibration source when the UTM is running, and the vibration on the specimens caused by the hydraulic pump can bring the noises into AE monitoring. The vibration on the specimens may vary with the structures of the test machines and the damage degree of the specimen, according to the reference [29]. Schiavi and his colleagues [30] observed that abundant AE signals and mechanical noises were generated at the frequency band of 0~10 kHz in uniaxial compression tests done in a hydraulic UTM. Additionally, the AE noises were also observed in our pretests conducted in a hydraulic UTM (depicted in Figure 1) and the AE noises were in the low frequency band (0~100 kHz), or with the low amplitude (less than 10 mV, which is equal to 40 dB under the amplification of 40 dB). For eliminating the potential background noises or being limited by the frequency response characteristics of experimental instruments, many researchers [31,32,33,34] raised the threshold or filtered out the signals at the low frequency band, which would result in the loss of some important signals in tests. Hence, it is necessary to eliminate the AE noises with less loss of useful AE data in the DTT before applying the AE technology.

In the research for this paper, a novel thermal tensile testing machine (TTTM) was constructed. The AE technology was applied to monitor the damage process of plain concrete in the DDTs conducted on the TTTM. The TTTM was powered by the thermal expansion of loading columns, and had a stiffness similar to the specimen, thus avoiding the influence of potential AE noises and simulating the rapid fracture process in real concrete structures. Meanwhile, due to the sudden change of deformation and load at the rapid fracture process, a static-dynamic acquisition system was developed, which could switch the acquisition mode from the static mode to the dynamic mode automatically. Some complete tensile stress-strain curves were obtained with the developed system, and the damage evolution of plain concrete in DTTs was analyzed.

## 2. Experiment

### 2.1. Materials, Mix and Specimens

The mix proportion of concrete is given in Table 1. The Type 42.5R Portland cement (Xinning New Building Materials Co., Nanjing, Jiangsu, China) was used in the mix. Crushed limestone with a maximum aggregate size of 20 mm was used as a coarse aggregate, and the maximum size of sand grains was 4 mm.

The dimension of specimens was 100 mm × 100 mm × 300 mm. After the casting and hardening of the concrete, the specimens were covered with a plastic membrane to prevent moisture from evaporating. The specimens were demolded 24 h later, and then cured by being sprayed with water daily and covered with the plastic membrane until the end of a 28-day period. These specimens were stored in an indoor dry environment after the curing, and tested a year later. Before testing, the specimens were processed, which will be elaborated upon in Section 2.5**.**

### 2.2. Experiment Instruments

**Figure 2:**
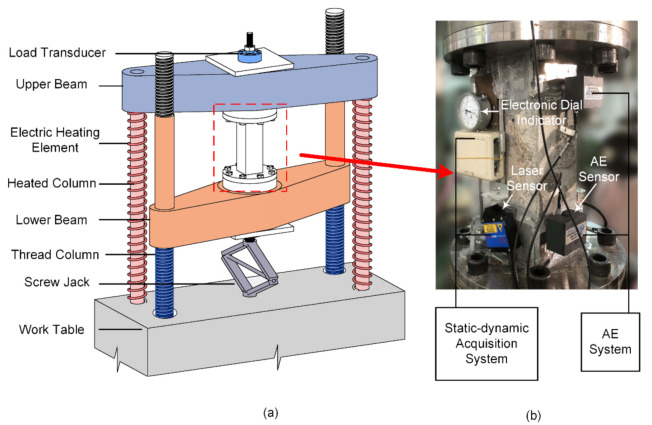
Schematic diagrams of: (**a**) the modified tensile testing machine; and (**b**) acquisition systems.

The direct tension test was conducted on the TTTM, which was reformed from a conventional hydraulic UTM (WE-50, Changchun Testing Machine Factory, Changchun, Jilin, China). The schematic diagram of the TTTM is drawn as Figure 2a, and the detail and operation steps of the TTTM will be expounded below. An eight-channel PCI-2-based AE system produced by Physical Acoustics Corporation (Princeton Junction, NJ, USA) was used in the tests, and the AEwin system was adopted to analyze the data. Four R15 AE sensors (whose resonance frequency is 150 kHz) were put on the two opposite sides of the specimen diagonally to locate the AE signals (shown in Figure 2b). In view of the frequency response characteristic of the R15 AE sensor and the level of background noise, the threshold of the AE acquisition system was set as 30 dB, and the filtering spectrum was set as 20 kHz~1000 kHz. For the reason that the rapid failure process at the post-peak stage would cause large variations of deformation and load at a very short time (duration was about 4 ms), two optoNcDT LD1610 laser displacement sensors (Micro-Epsilon Messtechnik GmbH & Co. KG, Ortenburg, Niedersachsen, Germany) were installed on the other two sides of the specimen for the dynamic deformation data (shown in Figure 2b). Meanwhile, to ensure the acquisition accuracy of the deformation data before the fracture, two electronic dial indicators were placed beside the laser displacement sensors. The electronic dial indicators were reformed from the dial indicators by adding the deformation-to-voltage conversion devices, and the measuring accuracy could be ensured by comparing the recorded data with the readings on the dial indicators. The load transducer was a spoke-type pressure sensor with the measuring range of 0~50 kN. Avoiding data redundancy, the sampling frequency for deformation and load was 5 Hz at the static acquisition mode and 100 kHz at the dynamic acquisition mode, respectively.

### 2.3. Thermal Tensile Testing Machine

The loading process on the original UTM was realized by the hydraulic system before reforming. However, the hydraulic system might generate mechanical and electric noises during the AE monitoring, and even some important AE signals could be flooded by the AE noises. For this reason, the UTM was reformed to achieve silent operation by the thermal loading system. The reformed UTM, i.e., the TTTM, had four columns and two beams. Two threaded columns could be controlled to rotate clockwise or anticlockwise to move the lower beam up or down, and the other two columns would be heated by electric heating elements during the loading process. The two heated columns elongated under thermal expansion, so that the upper beam would be elevated and the specimen could be in tension through the load-transferring device.

**Figure 3:**
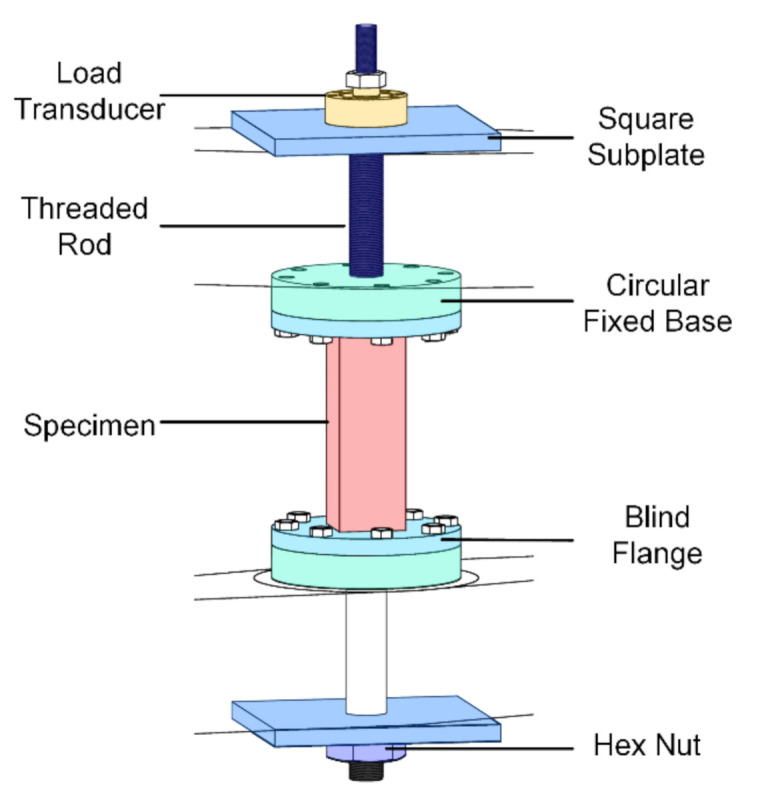
The schematic diagram of load transferring device.

The original holding device of the UTM was replaced with a load-transferring device, as shown in Figure 3. The load-transferring device included two blind flanges, two circular fixed bases, two square subplates, two threaded rods, two hex nuts and dozens of bolts. The blind flanges were designed to provide replaceable bases for sticking to the specimen, thus making it convenient to install and remove specimens. The circular fixed bases were connected with the threaded rods by threads, and the blind flanges were fixed on the circular fixed bases with bolts during the tests.

The process of heating columns was realized by connecting the electrically-heated coils winded around the columns with current. Accordingly, the deformation rate of the specimen could be controlled roughly through adjusting the current in the electrically-heated coils. The diameter and the length of the heated columns are 70 mm and 1000 mm, respectively. In view of the heating range of the electrically-heated coils and the actual efficiency of heat transference, the max amplitude of the temperature rise on the columns could only be 120 °C. The material of columns is steel and its thermal expansion coefficient is 1.2 × 10^−5^/°C, therefore the max elongation of the heated columns is just 1.4 mm. Even though the value exceeds the max deformation of specimens at the tensile failure moment (if the nominal strain of specimens at tensile failure moment was presumed to be 1000 με, the corresponding deformation of specimens would be 0.3 mm), the max elongation would still be insufficient if too much clearance existed among the components on the load transfer path. For this reason, the clearance among the components was reduced as much as possible before the heating process. The thread clearance between the thread columns and lower beam was reduced by elevating the lower beam with the screw jack below, and other clearance was reduced by the preloading of nuts and bolts. The nut on the load transducer was the last one to preload, and the last preloading process should be carried out under load monitoring, as the process would put the specimen under certain tension.

### 2.4. Stiffness of Thermal Tensile Testing Machine

**Figure 4:**
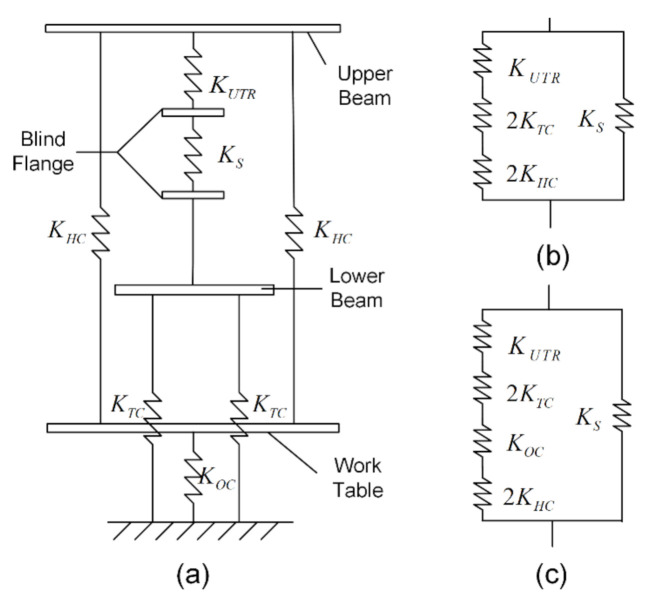
Stiffness calculation diagrams of testing machines: (**a**) stiffness schematic of components; and the stiffness calculation models of (**b**) the thermal tensile testing machine (TTTM); and (**c**) the original UTM.

In view that the stiffness of the test machine has a certain influence on the test result of the post-peak part, the stiffness comparison between the TTTM and the original UTM is presented in this section. The main difference between the two test machines is the load power system, which can change the load transfer path and determine whether to consider the stiffness of the oil cylinder in the stiffness calculation. Without regard to the influences of some components (such as beams, the load transducer and threaded connections) on the stiffness calculation, the stiffness schematic of components can be depicted as Figure 4a, and the stiffness value of each component is also calculated and presented in Table 2. The released energy of the lower thread rod and bolts at the fracture moment is negligibly small (due to the preloading before testing), so their stiffness is not considered in the calculation models. In the stiffness calculation model of the TTTM (shown as Figure 4b), the stiffness of the oil cylinder is large enough to be deemed as a rigid body, due to the piston part of the oil cylinder having fallen to the bottom before testing. The stiffness of the TTTM can be obtained from Equation (1), and the value is 520.76 kN/mm. However, in the stiffness calculation model of the original UTM (shown as Figure 4c), the oil cylinder is considered as an elastic body. The stiffness of the original UTM can be obtained from Equation (2), and the value is 39.22 kN/mm. According to the calculations, the stiffness of the TTTM is close to the specimen, while the corresponding value of the UTM is much smaller than the specimen. Therefore, the elastic energy stored in the UTM would be much more than the one stored in the TTTM when DTTs were conducted on the UTM and the TTTM, respectively, which could result in the macro-cracks of specimens tested on the UTM occurring earlier and developing faster than those tested on the TTTM.

(1) $$K_{TTTM}=\frac{1}{1/K_{UTR}+1/\left(2K_{TC}\right)+1/\left(2K_{HC}\right)}$$

(2) $$K_{UTM}=\frac{1}{1/K_{UTR}+1/\left(2K_{TC}\right)+1/\left(2K_{HC}\right)+1/K_{OC}}$$

### 2.5. Experimental Procedure

The whole test process can be simplified as seven steps, which are presented and elaborated below.

**Figure 5:**
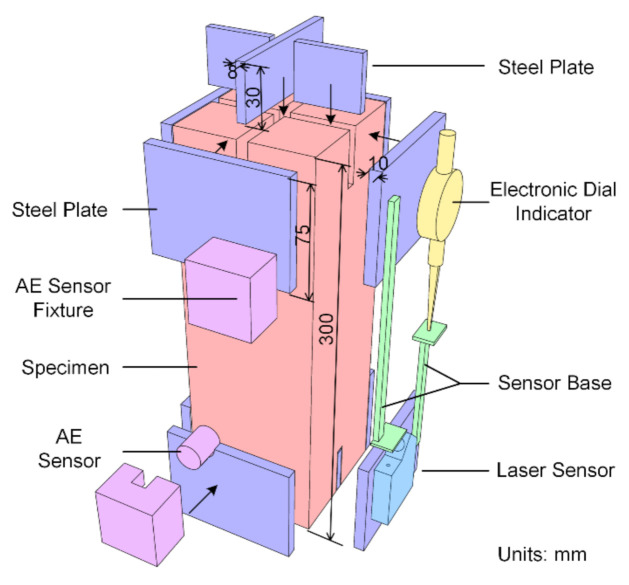
The diagram of specimen processing and sensor installation.

1. Processing the specimen. To ensure uniform stress distribution at the ends of the specimen, the sticky steel glue with high strength (which is usually applied in the structure strengthening field, and whose tensile strength can reach 30 MPa) was used to stick the specimen on the blind flanges. Meanwhile, to avoid fractures near the ends of the specimen or at the interfaces between the specimen and the blind flanges, the specimen was reinforced with steel plates inside and around the ends of the specimen (shown as Figure 5), and the end surfaces of the specimen were polished to remove the low-strength laitance layer. The detailed reinforcing process is described as follows: two crossed square grooves were slotted on the ends of the specimen (each groove was 1 cm wide and 3 cm deep), and the corresponding steel plates were then put in the grooves and around the ends of the specimen with the glue after removing the dust on the surfaces.
2. Preloading bolts and nuts. Before the specimen was stuck on the blind flanges, the blots on the blind flanges and the nuts on the thread rods were preloaded. The step could make the load-transferring device and beams become an entirety, thus weakening the influence of the load-transferring device on the stiffness of the TTTM and minimizing the disturbance of the specimen during and after the sticking process.
3. Sticking the specimen. After the glue applied at the processing step became solidified, the specimen was placed on the TTTM with adequate glue on the ends of the specimen. Furthermore, the surfaces of the specimen and blind flanges were wiped with alcohol before sticking. Then, the power of the test machine was turned on and the lower beam was elevated by the rotation of thread columns until the redundant glue on the ends of the specimen came out, thus minimizing the thickness of the glue layer and clearance among components.
4. Installing sensors. The sticky steel glue needs at least three days to reach the designed strength, so the installation of sensors (shown in Figure 5) could be done during this period. The measurement area for obtaining the deformation was the middle section of the specimen with the length of 150 mm. The laser displacement sensors and the electronic dial indicators were bonded on the steel reinforcement plates. Four AE sensors were attached on the specimen with fixtures, making the coupling layer between AE sensors and the specimen thinner and more compact.
5. Reducing the clearance. The nut on the load transducer was first released, and then the lower beam was lowered about 1 cm for the space needed in the next steps. After that, the lower beam was elevated by the screw jack to reduce the thread clearance between the thread columns and the lower beam. Finally, the nut on the load transducer was preloaded to reduce the clearance among the upper subplate, the load transducer and the upper threaded rod. Meanwhile, there is the last preloading which needs to be done under load monitoring, in which the load should be below 2 kN (about 10% of peak load).
6. Heating the columns. The electrically-heated coils wound around the columns were connected with the current after the previous steps, and instruments started to record data at the same time. To adjust the deformation rate, a transformer was connected with electrically-heated coils in series.
7. Switching acquisition mode. To avoid missing the deformation and load data at the instantaneous fracture process, two methods were set to switch the acquisition mode from the static mode to the dynamic mode. The first method was to switch the acquisition mode automatically according to the abrupt fall of the load rate, and the second one was to switch when abundant AE signals were generated around the peak stress level. Additionally, the latter could only be done manually, as the AE acquisition system was independent with the acquisition system of deformation and load in the tests.

## 3. Results and Discussions

### 3.1. Load Rate and Deformation Rate of the TTTM

**Figure 6:**
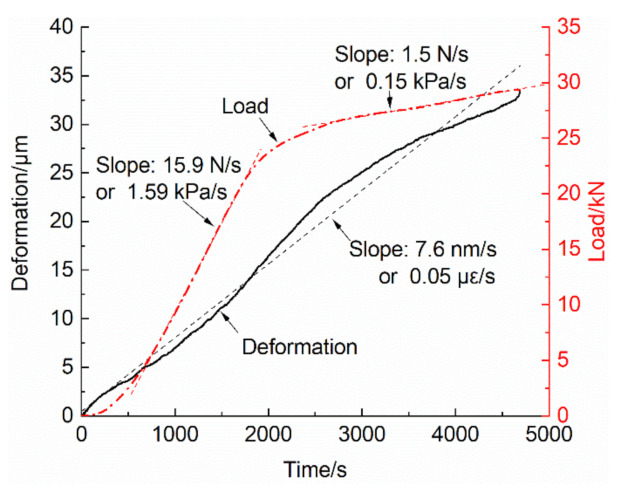
The diagram of deformation and load vs. time.

The performance of the TTTM can be observed from the diagram of deformation vs. time before the fracture moment (shown as Figure 6), in which the deformation value is the average of data acquired by two electronic dial indicators. It can be seen from Figure 6 that the deformation of measurement area is approximately linear, and its slope is about 7.6 nm/s, or 0.05 με/s. The tortuosity of the deformation vs. time curve may be attributed to the internal force redistribution of components or the data missing from the deformation on the other two sides of the specimen. Contrary to the deformation data, the load rate varies from 15.9 N/s to 1.5 N/s. The load process at the pre-peak stage can be roughly divided into two phases according to the load rate vs. time curve, in which the initial part and the connecting part between the two phases are not considered. The first phase is related to the linear elastic stage of concrete, and the second is related to the non-linear stage. Due to the extremely short duration of the fracture process (which is about 4 ms), the load rate and the deformation rate during this process are much larger than those at the previous time.

### 3.2. Static-Dynamic Acquisition Switching System

Owing to the sudden change of load and deformation at the instantaneous fracture, the data at this stage were obtained by the dynamic acquisition system, but the data redundancy would emerge if the dynamic acquisition lasted too long. Meanwhile, the condition of specimens is not stable around the peak stress level, and they may fracture at any moment. For these reasons, the acquisition system needs to be switched automatically from the static mode to the dynamic mode near the fracture moment. The switching moment should be less than 30 s and more than 5 s before the fracture moment, so that the stored data would not be too large and the data during the fracture process could also be acquired. After the test, the data at the fracture process could be extracted from the acquired data.

**Figure 7:**
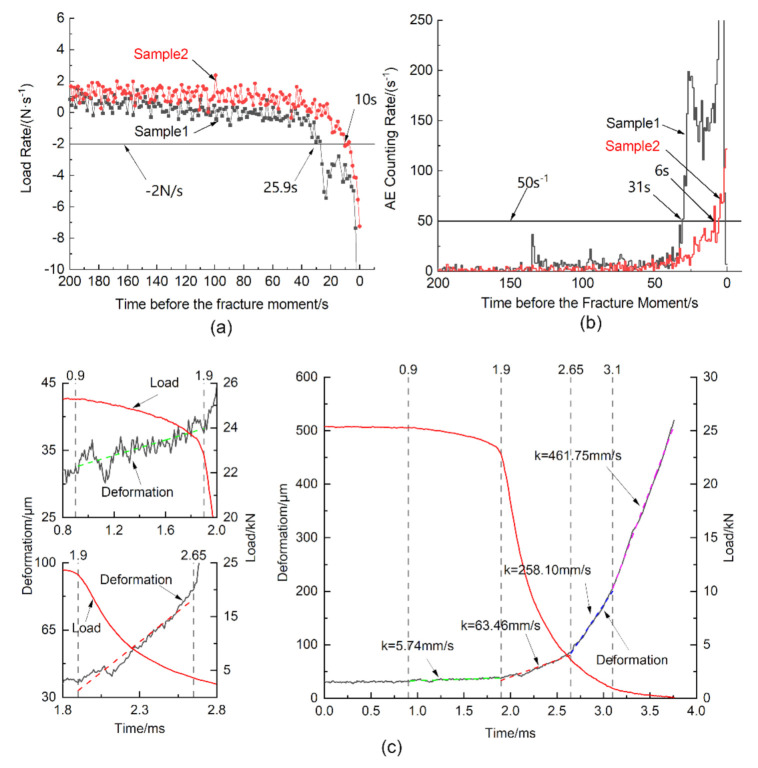
Diagrams of: (**a**) load rate vs. time; and (**b**) AE counting rate vs. time before the fracture moment; as well as (**c**) deformation and load vs. time during the fracture process.

Compared with load signals, deformation signals might not be sensitive enough to determine the switching moment timely, and the trends of deformation on two sides of the specimen might not be consistent around the peak stress level (as the deformation signals on different sides of the specimen could be affected by the potential eccentric loading). According to the test data, the switching moment was set as the moment when the load rate was less than −2 N/s and the load was larger than 20 kN at the same time (to avoid switching the acquisition mode at the unstable initial loading stage). Figure 7a shows the curve of load rate vs. time, in which the load rate keeps constant basically during 200~50 s before the fracture moment and then gradually drops below −2 N/s. Except for the load rate, the AE counting rate is also a great indicator to determine the switching moment, which is obvious in Figure 7b. However, two systems are independent at present and the real-time display of AE is a little delayed, so switching according to the AE counting rate can only be an assistant method to ensure that the acquisition mode was switched in time. The deformation and load data during the sudden fracture process can be extracted from the stored data after tests, and the extracted data of a sample is showed as Figure 7c, in which the deformation is the average of data acquired by two laser displacement sensors. During the fracture process, the released elastic energy stored in the TTTM and the specimen before fracture caused a huge growth in the strain rate, which varied from 10^−5^/s to 10^0^/s. According to the variations of the deformation rate, the curves at this stage can be divided into four phases, which may correspond to the generation of numerous micro-cracks, the growth of macro-cracks, the rapid development of macro-cracks and the final fracture of the specimen. As shown in Figure 7c, the deformation rate at each phase is different, especially at the last two phases (where the deformation increases much faster than that at the first two phases). The reason for this may be that the crack process was transformed from the stable cracking to unstable cracking, and the energy stored in the load system before the fracture moment was released rapidly at those phases.

### 3.3. Failure Mode of Specimens

The boundary conditions of specimens under direct tension can affect the crack propagation around and after the stress peak [3,25,35]. The boundary conditions of the specimens tested on conventional UTMs are usually rotating-rotating or fixed-fixed, which are different from the boundary conditions of the specimens on the TTTM. Considering the space between the top circular fixed base and the upper beam (which is about 1 cm wide), the top circular fixed base could be rotated to a certain degree at all directions. For this, the fixture at the top end of the specimen could be considered as an approximate spherical hinge. Meanwhile, the preload procedure made the bottom circular fixed base cling to the lower beam without any rotation possible, and the boundary conditions at the bottom end of the specimen could be considered as clamped support. In other words, the boundary conditions of the specimens on the TTTM were fixed-rotating, which could serve as a compromise to the issues associated with the fixed-fixed and the rotating-rotating boundary conditions [25].

**Figure 8:**
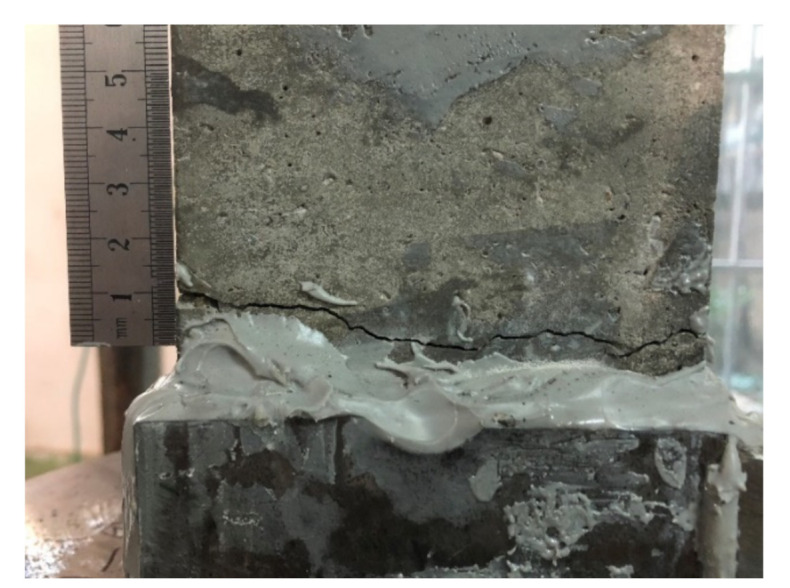
The crack at the end of a specimen.

**Figure 9:**
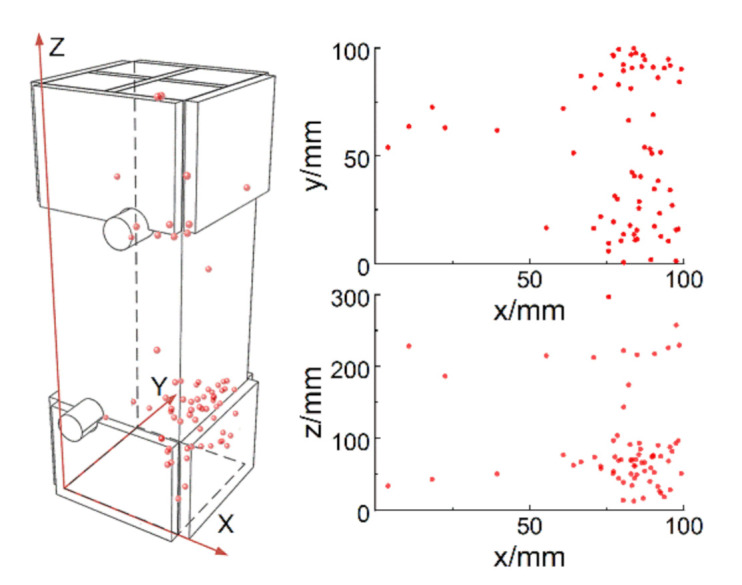
The AE locations of the specimen under eccentric loading.

The stress distribution inside the specimen is nonuniform for the inhomogeneity of concrete material, which includes initial micro-cracks, voids and randomly distributed coarse aggregates. The cracks would occur and propagate at the weakest region of the specimen, so eccentric loading could hardly be avoided during the test, and the final fracture might occur randomly on the unreinforced area. The fracture location of most specimens was near the ends of the specimens (shown as Figure 8), and the propagation of cracks was not observed as the cracks were almost only visible near the fracture moment. The phenomenon of eccentric loading, however, can be demonstrated by the AE locations. Figure 9 shows the AE locations of a specimen under the eccentric loading, in which the locations of AE signals are concentrated on one side of the fracture plane and the phenomenon is obvious in the x-y plane (i.e., micro-cracks emerged intensively in those regions before the fracture moment). The crack near the specimen ends might be caused by the steel plates stuck on the sides of the specimen ends. The steel plates affect the stress filed during the tension, resulting in that the stress near the margin of the steel plates is a little higher than the stress at the middle section of the specimen. This phenomenon is common in the DTTs [25], but the influence on tensile properties is little.

### 3.4. Stress-Strain Curves

**Figure 10:**
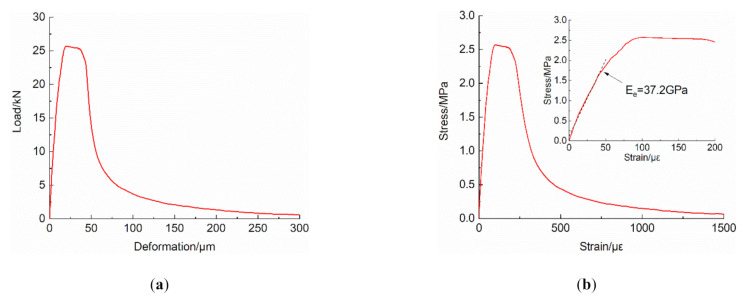
Curves of: (**a**) load vs. deformation; and (**b**) stress vs. strain.

The complete load vs. deformation curve can be obtained through splicing the data recorded by the static acquisition system and the dynamic acquisition system, respectively, which is depicted in Figure 10a. Assuming that the macro-cracks generated around the peak stress level, the post-peak strain could be revised by Equation (3) to eliminate the effect of elastic shrinkage in uncracked regions [2]. Consequently, the stress-strain curve can be transformed from the load vs. deformation curve (shown as Figure 10b)

(3) $$\varepsilon_{post-peak}=\delta /l+\left(\sigma_{p}-\sigma \right)/E_{e}$$

where  δ is the deformation of the measurement area, *l* is the length of the measurement area, σ is the nominal stress, $\sigma_{p}$ is the peak stress and $E_{e}$ is the elastic modulus obtained by fitting the stress-strain data at the linear elastic stage.

**Figure 11:**
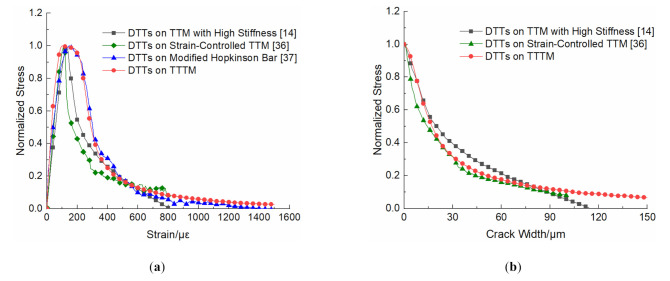
Curves of: (**a**) normalized stress vs. strain; and (**b**) normalized stress vs. crack width obtained from different test machines.

To compare the test results of the DTTs conducted on different test machines, the complete stress-strain curves were extracted from the relevant literatures [14,36,37]. As shown in Figure 11a, the stress values in each curve were normalized for a comparative analysis of curves shapes. The DTTs on the TTM with high stiffness [14] are realized by introducing two steel rods parallel to the specimen at the force transferring path, and the strain-controlled UTM [36] refers to the closed-loop controlled hydraulic TTM, which can load by controlling the deformation on the specimen in a timely manner. The DTTs on the modified Hopkinson bar [37] are used to describe the dynamic tension behavior of concrete at strain rates of 10^0^/s, which are closed to the strain rates of tests on the TTTM at the post-peak stage. It is obvious in Figure 11a that the curves are basically the same at the pre-peak stage, and the main differences concentrate at the post-peak stage. At the beginning of the post-peak stage, the curves of the DTTs on the strain-controlled TTM and TTM with high stiffness drop faster, while the corresponding curves of the modified Hopkinson bar and the TTTM drop slower, and almost at the same rate. Those differences may be attributed to the real strain rates on the specimen near the dropping section, and the larger strain rates can result in slower drop rates, according to the strain rate effect. The reason that the DTTs on strain-controlled TTM drop at the fastest rate could be that the machine can control the strain rate stably without large energy released instantaneously, which is difficult for the rest of the test machines. However, the tensile fracture process of concrete on strain-controlled TTM con not describe the tensile fracture process of concrete structures comprehensively, especially when the energy released by concrete structures is large enough to trigger an unstable cracking process. The test results on the TTTM can better describe the tensile behavior of concrete materials in the real structures when unstable cracking process occurs.

To further verify the validity of TTTM, the normalized stress vs. crack width curve of tests on the TTTM is compared with that in available literatures [14,36] (as shown in Figure 11b). To make the comparison easier, the data of DTTs on the TTTM is obtained by analyzing the second and third phase of the post-peak data (i.e., the data of 1.9–3.1 ms in Figure 7c). The first phase corresponds to the generation of numerous micro-cracks, which is unsuitable and difficult to transform the deformation to crack width. The crack width in Figure 11b is calculated by Equation (4) to eliminate the effect of elastic shrinkage in uncracked regions of specimens [2]

(4) $$w=\delta -\delta_{p}+\left(\sigma_{p}-\sigma \right)l/E_{e}$$

where $\delta_{p}$ is the peak deformation of the measurement area. It can be observed from Figure 11b that the curves are basically same, which demonstrates the validity of TTTM.

In addition, the rapidly changing deformation and load at the post-peak phase are susceptible to the experimental conditions. The filter cutoff frequency of acquisition instruments and the performances of sensors at high sampling frequency would both affect the accuracy of data at the post-peak phase.

### 3.5. AE Monitoring Analysis

Due to the extremely short duration of the fracture process, the AE data at this process was very limited. Fortunately, the AE data before the fracture moment was plenty, and numerous signals increased with the loading time.

**Figure 12:**
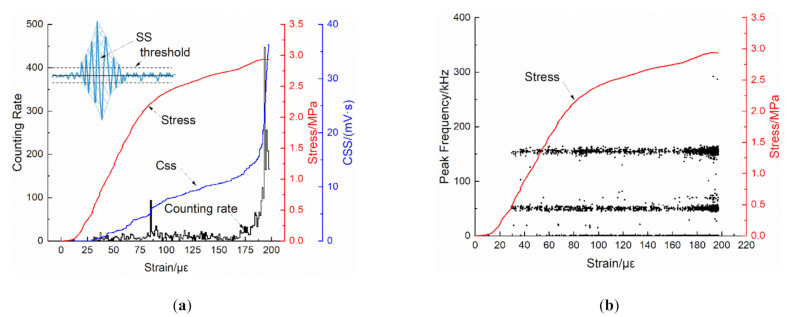
Diagrams of: (**a**) strain vs. counting rate and cumulative signal strength (CSS); and (**b**) peak frequency vs. strain.

The signal strength (SS) is the area inside the envelope curve of the AE waveform packet (the waveform of an AE signal, starting from the first time when the waveform amplitude exceeding the threshold and ending at the last time). The cumulative signal strength (CSS) is the accumulated value of the SS with the testing time, and is a useful indicator to analyze the concrete damage evolution. Figure 12 presents the AE monitoring results of a specimen before the fracture moment, in which the data is the integration of four channels by selecting the highest amplitude signal from signals of all channels generated at the same time (within a arrival time difference of 500 ns). The counting rate in Figure 12a is the number of AE events during every 1 με, and it has two peaks, of which one occurs in the transitional region from the linear elastic stage to the non-linear stage and another one is around the peak stress level. Meanwhile, the CSS curve grows slowly before the stress peak, but rises sharply around the peak stress level. Therefore, it could be concluded that some micro-cracks initiate at the linear elastic stage and propagate at the nonlinear stage, and then more micro-cracks initiate and propagate intensively around the peak stress level.

In addition, one important reason for designing the TTTM is to avoid the potential background noises emerging in the DTTs conducted on the conventional UTM. Among the AE parameters, the peak frequency is an important reflection on the frequency characteristics of signals, which is the corresponding frequency of the highest Fourier transform value of a signal waveform. The scatter diagram of peak frequency vs. strain is depicted in Figure 12b, in which lots of signals emerge at the low frequency band (<100 kHz) and several signals with high frequency (>250 kHz) occur around the peak stress level.

**Figure 13:**
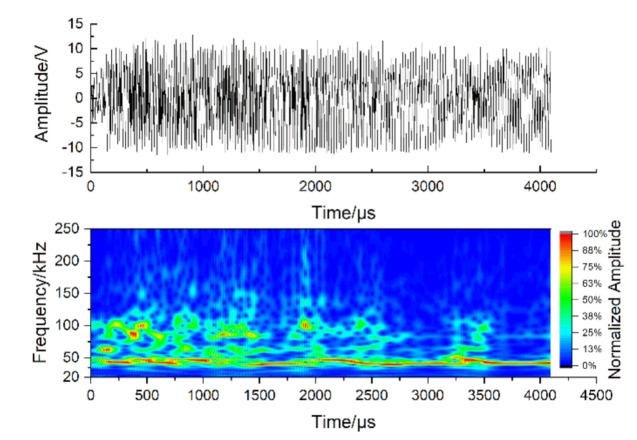
The waveform and frequency-time diagram of the AE signal at the fracture process.

As mentioned above, the number of AE signals at the fracture process is very limited, so it is almost impossible to analyze the fracture process of the specimen through AE parameters; the waveform analysis shown in Figure 13 is the only available way. The waveform data in Figure 13 is modified from the original data, in which the data points exceeding the limit of voltage acquisition (±10 V) are replaced with spline interpolations. The amplitude of the waveform is ultrahigh, and the duration of the signal is really long (compared with the signals at the pre-peak stage). Furthermore, the shape of the waveform approximates to be rectangular, which is different from the spindle shape of typical AE waveforms. All of these characteristics may be the result of longstanding intensive AE sources (i.e., the cracking process at the moment lasts longer or involves more crack initiations and propagations than the general cracking process at the pre-peak stage). The time-frequency analysis of the waveform shown in Figure 13 was done by the wavelet transform. It shows that the energy of the waveform is concentrated on the frequency bands of 20~50 kHz and 80~120 kHz, particularly at the front of the waveform.

## 4. Conclusions

To study the complete tensile stress-strain response of plain concrete, a TTTM for the direct tension test was developed by reforming a conventional hydraulic UTM, and its stiffness was close to the specimen. AE technology and a static-dynamic acquisition system were also applied in the tests. The main conclusions could be drawn as follows:

1. The complete tensile stress-strain curve was obtained from the direct tension test conducted on the TTTM, of which the data at the rapid fracture process was acquired by the dynamic acquisition instrument and laser displacement sensors. The performance of the TTTM and corresponding test procedures were introduced in detail.
2. With the developed TTTM, the mechanical response of concrete at the fracture process was studied. During the fracture process of the concrete in direct tension, the released elastic energy stored in the experimental machine system before fracture could cause a large variation of the strain rate, which varies from 10^−5^/s to 10^0^/s.
3. A static-dynamic acquisition system was established to deal with the sharp variations of the deformation and load at the post-peak stage. The acquisition mode of the acquisition instrument could be switched from the static mode to the dynamic mode automatically before the fracture moment. The phenomenon of the AE data explosion could also trigger the switching of the acquisition mode during tests.
4. The developed TTTM could avoid the potential AE noises emerged in tests conducted on the conventional UTM, and AE results of plain concrete tested on the TTTM show that large amounts of information at the low frequency band (<100 kHz) exist.
5. The AE technology is a useful approach to determine the fracture locations in real time, and provides an efficient method to study the damage evolution of plain concrete. With the measured AE data, it could be concluded that some micro cracks initiate at the linear elastic stage of concrete in direct tension and propagate at the nonlinear stage, and then more micro-cracks occur intensively in concrete around the peak stress level.

## Figures and Tables

**Table 1:** Details of the mix proportion (in per cubic meter of concrete).

Cement (kg)	Water (kg)	Coarse Aggregate (kg)	Fine Aggregate (kg)
420	168	745	1177

**Table 2:** Stiffness calculations of components.

Components	Length (mm)	Sectional Area (mm^2^)	Elastic Modulus (MPa)	Stiffness (kN/mm)	
				Symbol	Value
Heated column	1000	3848	210,000	$K_{HC}$	808.17
Thread column	800	3318	210,000	$K_{TC}$	871.06
Upper thread rod	300	1963	210,000	$K_{UTR}$	1374.45
Specimen	300	10,000	30,000	$K_{S}$	1000.00
Oil cylinder *	300	6362	2000	$K_{OC}$	42.41

* In the stiffness calculation, the oil cylinder is simplified as a compressed cylinder with the elastic modulus transformed from the bulk modulus of hydraulic oil.

## Data Availability

Data Sharing is not applicable.
